# Improved Spatial Differencing Scheme for 2-D DOA Estimation of Coherent Signals with Uniform Rectangular Arrays

**DOI:** 10.3390/s17091956

**Published:** 2017-08-24

**Authors:** Junpeng Shi, Guoping Hu, Fenggang Sun, Binfeng Zong, Xin Wang

**Affiliations:** 1 Air and Missile Defense College, Air Force Engineering University, Xi’an 710051, China; 15667081720@163.com; 2 College of Information Science and Engineering, Shandong Agricultural University, Tai’an 271018, China; sunfg@sdau.edu.cn; 3 Unit-94710 of the PLA, Wuxi 214000, China; zongbinfeng@163.com; 4 Unit-94259 of the PLA, Penglai 265600, China; 18615073747@163.com

**Keywords:** improved spatial differencing, two-dimensional direction of arrival estimation, uniform rectangular arrays, difference-operation, sample covariance matrix

## Abstract

This paper proposes an improved spatial differencing (ISD) scheme for two-dimensional direction of arrival (2-D DOA) estimation of coherent signals with uniform rectangular arrays (URAs). We first divide the URA into a number of row rectangular subarrays. Then, by extracting all the data information of each subarray, we only perform difference-operation on the auto-correlations, while the cross-correlations are kept unchanged. Using the reconstructed submatrices, both the forward only ISD (FO-ISD) and forward backward ISD (FB-ISD) methods are developed under the proposed scheme. Compared with the existing spatial smoothing techniques, the proposed scheme can use more data information of the sample covariance matrix and also suppress the effect of additive noise more effectively. Simulation results show that both FO-ISD and FB-ISD can improve the estimation performance largely as compared to the others, in white or colored noise conditions.

## 1. Introduction

Two-dimensional direction of arrival (2-D DOA) (i.e., elevation and azimuth angles) estimation of multiple signals with different array geometries is an important problem in many practical applications such as radars and wireless communications. Various methods have been developed for solving this problem [1,2,3,4,5,6,7,8,9,10], such as the subspace-based methods [1,2,3,4,5,6], the sparse reconstruction methods [7,8,9], and the least-square approach [10].

However, for coherent signals [11,12,13], the traditional methods, such as the multiple signal classification (MUSIC) [14] and estimation of signal parameters via rotational invariance technique (ESPRIT) [15], suffer from performance degradation due to the rank-deficiency of the signal covariance matrix. The forward backward spatial smoothing (FBSS) technique [16] is very effective in removing the coherency but at the cost of few degrees of freedom (DOFs). Therefore, spatial smoothing techniques with URAs are developed by using virtual sensors to increase the DOFs, e.g., the unitary ESPRIT [17] and spatial smoothing MUSIC [18] algorithms. The 2-D spatial smoothing methods were also applied for DOA estimation [19] or joint DOA and direction of departure (DOD) estimation [20] with multi-input multi-output (MIMO) radar, where the transmission and reception diversity smoothing is derived by constructing a new covariance matrix with decorrelated signal subspace. Besides, compared with the higher-order cumulants methods [21,22], spatial smoothing methods also have a lower computational complexity.

Recently, spatial differencing techniques [23,24,25,26,27] have been introduced to suppress the effect of additive noise. Aiardi et al. [23] proposed a high-resolution DOA estimation method by performing the partial spatial differencing operation; the methods in [24,25] can suppress white noise or colored noise by using the difference between the first and backward subarrays or between the neighboring subarrays, respectively. Liu et al. [26] developed a generalized covariance differencing algorithm by using the difference between the FB smoothing matrix and its complex conjugation. In fact, these methods [23,24,25,26] cannot be directly applied for 2-D DOA estimation. Then, similar with the 2-D configuration, the method in [27] explored four kinds of smoothing techniques including the spatial difference smoothing (SDS), asymmetric SDS (A-SDS), transmit-receive diversity SDS (TRD-SDS), and asymmetric TRD-SDS (A-TRD-SDS) for coherent targets with MIMO radar.

Nevertheless, the aforementioned spatial differencing techniques haven’t fully explored the advantages of the URA. On the one hand, the involved spatial smoothing subarrays can only use part of the data information of the sample covariance matrix. On the other, the classical spatial differencing methods only focus on the suppression of additive noise and few ones consider the information loss caused by the difference-operation. Therefore, in this paper, we propose an improved spatial differencing (ISD) scheme for 2-D DOA estimation of coherent signals with URAs, where both the forward only ISD (FO-ISD) and forward backward ISD (FB-ISD) methods are developed. Simulation results show the usefulness of the proposed methods. For clarity, the main advantages are given as follows:

- Classic spatial differencing techniques only use the data information of overlapped smoothing subarrays, while FO-ISD and FB-ISD can extract all the data information of each row or column rectangle subarrays.
- Classic spatial differencing techniques perform difference-operation on the whole overlapping subarrays, while FO-ISD and FB-ISD calculate the differencing matrix for the auto-correlations and keep the cross-correlations unchanged. So SD-SMS has less information loss, resulting in a more effective noise suppression.
- FB-ISD can achieve a further improved performance than FO-ISD due to the increased number of smoothing submatrices.

The rest of the paper is listed as follows. We first introduce the basic signal model of URA for coherent signals in Section 2. In Section 3, we develop the FO-ISD and FB-ISD methods by using the row or column rectangular subarrays, where the Cramér-Rao bound (CRB) is also given. Simulation results are presented in Section 4.1 and we conclude this paper in Section 5.

In this paper, operators ${\left(\cdot \right)}^{T}$, ${\left(\cdot \right)}^{*}$, and ${\left(\cdot \right)}^{H}$ represent transpose, conjugation, and conjugate transpose, respectively. $I_{N}$ denotes an N×N identity matrix and $J_{M}$ denotes an M×M exchange matrix with ones on its anti-diagonal and zeros elsewhere. ∘ and ⊕ represent the Khatri-Rao product and Hadamard product, respectively; diag(·) and blkdiag(·) denote the diagonal matrix or block diagonal matrix operator. E[·] and vec(·) denote expectation and vectorization, respectively.

## 2. System Model

**Figure 1:**
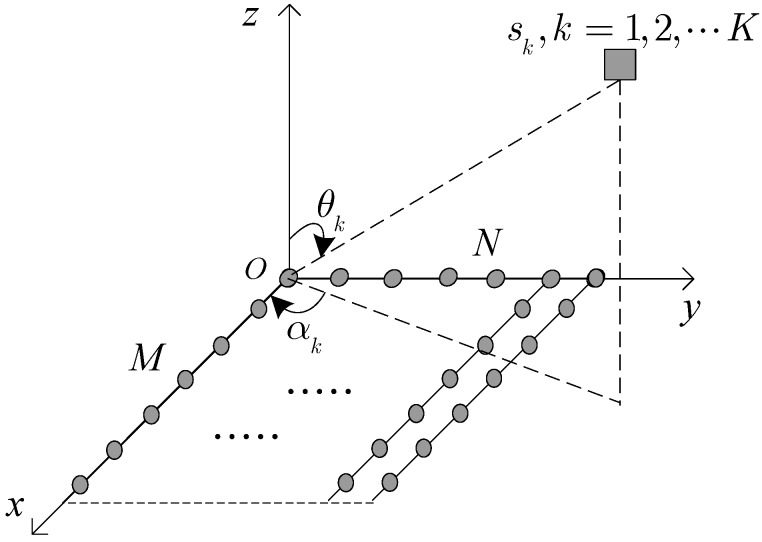
The geometry model of a URA.

As described in Figure 1, we consider *K* narrowband far-field coherent signals $s_{k}\left(t\right)$ (k=1,2,⋯,K) impinging on a URA (M×N sensors). We assume both *x* and *y* directions of the URA are separated by half a wavelength. Then the output can be expressed as [11]

(1) $$\begin{array}{c} X\left(t\right)=\sum_{k=1}^{K}a_{x}\left(\alpha_{k},\theta_{k}\right)a_{y}^{T}\left(\alpha_{k},\theta_{k}\right)s_{k}\left(t\right)+Z\left(t\right), \end{array}$$

where $\theta_{k}$ and $\alpha_{k}$ are the elevation and azimuth angles of the *k*-th signal; $a_{x}\left(\alpha_{k},\theta_{k}\right)=a_{x}\left(u_{k}\right)={\left[1,e^{-j\pi u_{k}},\ldots ,e^{-j\pi \left(M-1\right)u_{k}}\right]}^{T}$, $a_{y}\left(\alpha_{k},\theta_{k}\right)=a_{y}\left(v_{k}\right)=[1,e^{-j\pi v_{k}},\ldots ,e^{-j\pi \left(N-1\right)v_{k}}{]}^{T}$, $u_{k}=\sin\theta_{k}\cos\alpha_{k}$, $v_{k}=\sin\theta_{k}\sin\alpha_{k}$. The elements of Z(t) are temporally and spatially complex white Gaussian noises with zero-mean and variance $\sigma^{2}$. Then, vectorizing X(t) yields

(2) $$\begin{array}{c} x\left(t\right)=vec\left(X\left(t\right)\right)=\left(A_{x}\circ A_{y}\right)s\left(t\right)+z\left(t\right), \end{array}$$

where $A_{x}={\left[a_{x}\left(u_{1}\right),\cdots ,a_{x}\left(u_{K}\right)\right]}^{T}$, $A_{y}={\left[a_{y}\left(v_{1}\right),\cdots ,a_{y}\left(v_{K}\right)\right]}^{T}$, $s\left(t\right)={\left[s_{1}\left(t\right),\cdots ,s_{K}\left(t\right)\right]}_{K\times 1}^{T}$, and z(t) = vec(Z(t)). With *L* snapshots, we calculate the sample covariance matrix as

(3) $$\begin{array}{c} R_{0}=E[x\left(t\right)x^{H}\left(t\right)]=\frac{1}{L}\sum_{t=1}^{L}x\left(t\right)x^{H}\left(t\right)=AR_{s}A^{H}+\sigma^{2}I_{MN}. \end{array}$$

where t=1,2,⋯,L, $A=A_{x}\circ A_{y}$, and $R_{s}=E\left[s\left(t\right)s^{H}\left(t\right)\right]$ represents the signal covariance matrix.

## 3. 2-D DOA Estimation with URA

In this section, we firstly review the classic spatial differencing technique in [26] and then we derive the FO-ISD and FB-ISD methods in detail.

### 3.1. Classic Spatial Differencing Technique

The main idea of the classic spatial differencing technique is to build $Q_{x}Q_{y}$ overlapping rectangular subarrays with size of $P_{x}\times P_{y}$, i.e., sliding windows, where $Q_{x}$ and $Q_{y}$ are the forward subarrays along the *x* and *y* directions, respectively, $Q_{x}=M-P_{x}+1$, $Q_{y}=N-P_{y}+1$. Then, we can get the $\left(q_{x},q_{y}\right)$-th sliding window as

(4) $$\begin{array}{c} x_{q_{x}q_{y}}\left(t\right)=A_{P}\Phi_{x}^{q_{x}-1}\Phi_{y}^{q_{y}-1}s\left(t\right)+z_{q_{x}q_{y}}\left(t\right), \end{array}$$

where $A_{P}=A_{P_{x}}\circ A_{P_{y}}$, $A_{P_{x}}$ is the submatrix of the array response matrix $A_{x}$ consisting of the first row $P_{x}$ and the submatrix $A_{P_{y}}$ of $A_{y}$ consisting of the first row $P_{y}$; $q_{x}=1,\cdots ,Q_{x}$, $q_{y}=1,\cdots ,Q_{y}$, $\Phi_{x}=diag$$\left[e^{-j\pi u_{1}},\cdots ,e^{-j\pi u_{K}}\right]$, $\Phi_{y}=diag\left[e^{-j\pi v_{1}},\cdots ,e^{-j\pi v_{K}}\right]$, and $z_{q_{x}q_{y}}\left(t\right)$ is the corresponding noise vector. Then the covariance submatrix of $x_{q_{x}q_{y}}\left(t\right)$ can be given as

(5) $$\begin{array}{c} R_{q_{x}q_{y}}=A_{P}\Phi_{x}^{q_{x}-1}\Phi_{y}^{q_{y}-1}R_{s}\Phi_{y}^{1-q_{y}}\Phi_{x}^{1-q_{x}}A_{P}^{H}+\sigma^{2}I_{P_{x}P_{y}}, \end{array}$$

Using the covariance submatrix in (5), we can build the $p_{a}$-th SDS (i.e., asymmetric SDS, A-SDS) matrix as [26]

(6) $$\begin{array}{c} R_{p_{a}}^{f}=\frac{1}{Q_{x}Q_{y}}\sum_{q_{x}=1}^{Q_{x}}\sum_{q_{y}=1}^{Q_{y}}R_{q_{x}q_{y}}-\left(\frac{1}{Q_{x}Q_{y}-p_{a}}\right)\sum_{q_{x}=1,q_{x}\notin m_{a}}^{Q_{x}}\sum_{q_{y}=1,q_{y}\notin n_{a}}^{Q_{y}}R_{q_{x}q_{y}}^{b}, \end{array}$$

where $m_{a}=\left[m_{a}^{1},m_{a}^{2},\cdots ,m_{a}^{p_{a}}\right]$, $m_{a}^{i}\in \left[1,2,\cdots ,Q_{x}\right]$, and $1\leq i\leq p_{a}$; $n_{a}=[n_{a}^{1},$$n_{a}^{2},\cdots ,n_{a}^{f_{a}}]$, $n_{a}^{j}\in \left[1,2,\cdots ,Q_{y}\right]$, $1\leq j\leq f_{a}$; $R_{q_{x}q_{y}}^{b}=J_{P_{x}P_{y}}R_{q_{x}q_{y}}^{*}J_{P_{x}P_{y}}$.

We can see that A-SDS exploits the asymmetric difference between the complete forward spatially smoothed matrix and incomplete backward spatially smoothed matrix, and the noise can be suppressed by the differencing matrices in (6). However, since the forward and backward smoothed matrices have similar data structures, the difference-operation will have great information loss and performance will decrease greatly in the low SNR condition.

### 3.2. Improved Spatial Differencing (ISD) Scheme

#### 3.2.1. Analysis for Row Rectangular Subarrays

**Figure 2:**
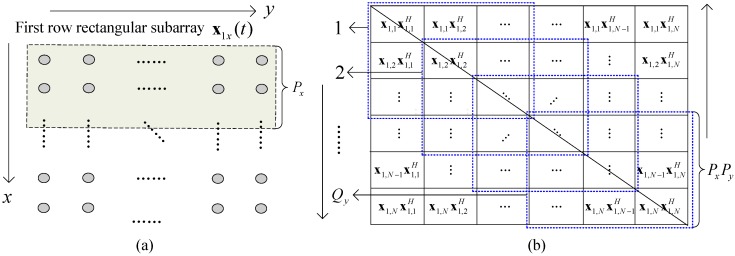
(**a**) The first row rectangular subarray (**b**) Its covariance matrix.

As described in Figure 2a, we divide the URA into $Q_{x}$ row rectangular subarrays along the *x* direction, where each one contains $Q_{y}$ sliding windows along the *y* direction. We take the first one as an example and write it as $x_{1x}\left(t\right)={\left[x_{1,1}^{T},\cdots ,x_{1,N}^{T}\right]}^{T}$, where $x_{1,n}$ denotes the first row $P_{x}$ of $x_{n}$, $x_{n}=A_{x}\Phi_{y}^{n-1}s\left(t\right)+z_{n}\left(t\right)$ is the received signal of the sensors in the *n*-th column of URA, n=1,⋯,N. Figure 2b describes the covariance matrix $R_{1x}$ of $x_{1x}\left(t\right)$. We can see that the data information within the blue box is constructed by the covariance submatrices $R_{1q_{y}}\left(t\right)$ ($q_{x}=1$) in (5). Therefore, in each row rectangular subarray, the sliding windows (within the blue box) can only use part of the data information of the covariance matrix $R_{mx}$, (m=1,2,⋯M), where $R_{mx}$ represents the covariance submatrix of $x_{mx}\left(t\right)$. To fully use this data information and also decrease the information loss caused by difference-operation, the FO-ISD and FB-ISD methods are given as follows.

#### 3.2.2. Forward only ISD (FO-ISD) Method

We continue to take the covariance matrix $R_{1x}$ in Figure 2b as an example. Since the matrix $R_{1x}$ is symmetric, we can only extract the submatrices below the diagonal ones from top to bottom. Each column submatrix of $R_{1x}$ can be divided into some column submatrices block, and the information of *n*-th $\left(n=1,\cdots ,Q_{y}-1\right)$ column can be set as

(7) $$R_{1,n}=\left\{\left[\begin{array}{c} x_{1,n}\\ x_{1,n+1}\\ \vdots \\ x_{1,n+P_{y}-1} \end{array}\right]x_{1,n}^{H},\cdots ,\left[\begin{array}{c} x_{1,Q_{y}}\\ x_{1,Q_{y}+1}\\ \vdots \\ x_{1,N}\; \end{array}\right]x_{1,n}^{H}\right\}=\left[\begin{array}{c} A_{P_{x}}\\ A_{P_{x}}\Phi_{y}\\ \vdots \\ A_{P_{x}}\Phi_{y}^{P_{y}-1} \end{array}\right]\Pi_{1,n}+\sigma^{2}C_{n}=A_{P}\Pi_{1,n}+\sigma^{2}C_{n},$$

where $x_{1,n}\left(t\right)=A_{P_{x}}\Phi_{y}^{n-1}s\left(t\right)+z_{1,n}\left(t\right)$, $z_{1,n}\left(t\right)$ is the corresponding noise vector with z(t); $\Pi_{1,n}=\left\{\Phi_{y}^{n-1}R_{s}\Phi_{y}^{1-n}A_{P_{x}}^{H},\Phi_{y}^{n}R_{s}\Phi_{y}^{1-n}A_{P_{x}}^{H},\right.\cdots ,\left.\Phi_{y}^{Q_{y}-1}R_{s}\Phi_{y}^{1-n}A_{P_{x}}^{H}\right\}$, and $C_{n}$$=\;[c_{1},0_{P_{x}P_{y}\times P_{x}},\cdots ,0_{P_{x}P_{y}\times P_{x}}]$, and $c_{1}=$${\left[I_{P_{x}},0_{P_{x}},\;\cdots ,0_{P_{x}}\right]}^{T}$. From (9), We observe that only the first submatrix contains auto-correlations, while the others are constructed from the cross-correlations. So we can perform the difference-operation on the first one, i.e.,

(8) $$\begin{array}{c} \left[\begin{array}{c} x_{1,1}\\ x_{1,2}\\ \vdots \\ x_{1,P_{y}} \end{array}\right]x_{1,1}^{H}-J_{P_{x}P_{y}}{\left\{\left[\begin{array}{c} x_{1,n}\\ x_{1,n+1}\\ \vdots \\ x_{1,n+P_{y}-1} \end{array}\right]x_{1,n}^{H}\right\}}^{*}J_{P_{x}}=A_{P}\left(R_{s}-\Theta^{*}\Phi_{y}^{1-n}R_{s}^{*}\Phi_{y}^{n-1}\Phi_{x}^{P_{x}-1}\right)A_{P_{x}}^{H}, \end{array}$$

where $J_{P_{x}P_{y}}A_{P}^{*}=A_{p}\Theta^{*}$, $\Theta =diag\{e^{-j\pi d_{1}},\cdots ,e^{-j\pi d_{K}}\}$, and $d_{k}=\left(P_{x}-1\right)u_{k}+\left(P_{y}-1\right)v_{k}$; $J_{P_{x}}A_{P_{x}}^{*}\;=\;A_{P_{x}}\Phi_{x}^{P_{x}-1}$. Using the differencing matrix in (8) to replace the first submatrix in (7), we have

(9) $$\begin{array}{c} {\bar{R}}_{1,n}=A_{P}{\bar{\Pi }}_{1,n}=A_{P}{\bar{H}}_{1,n}diag\left(A_{P_{x}}^{H},\cdots ,A_{P_{x}}^{H}\right). \end{array}$$

where ${\bar{H}}_{1,n}=\left\{\left(R_{s}-\Theta^{*}\Phi_{y}^{1-n}R_{s}^{*}\Phi_{y}^{n-1}\Phi_{x}^{P_{x}-1}\right),\cdots ,\Phi_{y}^{Q_{y}-1}R_{s}\Phi_{y}^{1-n}\right\}$, and the number of $A_{P_{x}}^{H}$ is $Q_{y}$ − n+1. We see that the matrix ${\bar{R}}_{1,n}$ can suppress the effect of noise by performing the difference-operation on the auto-correlations.

Then, the remaining $P_{y}$ columns of $R_{1x}$ (i.e., the last sliding window) can be expressed as

(10) $$\begin{array}{c} R_{1,Q_{y}}=A_{P}\Phi_{y}^{Q_{y}-1}R_{s}\Phi_{y}^{1-Q_{y}}A_{P}^{H}+\sigma^{2}I_{P_{x}P_{y}}, \end{array}$$

Likewise, performing the difference-operation on the matrix $R_{1,Q_{y}}$, we have

(11) $$\begin{array}{c} R_{1,Q_{y}}^{d}=R_{1,1}-J_{P_{x}P_{y}}R_{1,Q_{y}}^{*}J_{P_{x}P_{y}}=A_{P}\Pi_{1,Q_{y}}=A_{P}H_{1,Q_{y}}A_{P}^{H}, \end{array}$$

where $H_{1,Q_{y}}=\left(R_{s}-\Theta^{*}\Phi_{y}^{1-Q_{y}}R_{s}^{*}\Phi_{y}^{Q_{y}-1}\Theta \right)$.

Combining (9) and (11), the new differencing matrix constructed by the data information below the diagonal line can be rewritten as

(12) $$\begin{array}{cc} {\bar{R}}_{1} & =[{\bar{R}}_{1,1},{\bar{R}}_{1,2},\cdots ,{\bar{R}}_{1,Q_{y}-1},R_{1,Q_{y}}^{d}]\\ & =A_{P}\{{\bar{H}}_{1,1},{\bar{H}}_{1,2},\cdots ,{\bar{H}}_{1,Q_{y}-1},H_{1,Q_{y}}\}diag\left(A_{P_{x}}^{H},\cdots ,A_{P_{x}}^{H},A_{P}^{H}\right), \end{array}$$

where the number of $A_{P_{x}}^{H}$ is $\left(\left(Q_{y}+1\right)Q_{y}/2-1\right)$. Similar with the processing of the first row rectangular subarray, we can extract the information of the $q_{x}$-th subarray and form the corresponding differencing matrix $R_{q_{x}}$. As a result, the FO-ISD matrix can be defined as

(13) $$\begin{array}{c} R^{f}=\frac{1}{Q_{x}}\sum_{q_{x}=1}^{Q_{x}}R_{q_{x}}. \end{array}$$

where

(14) $$\begin{array}{c} {\bar{R}}_{q_{x}}=A_{P}\{{\bar{H}}_{q_{x},1},{\bar{H}}_{q_{x},2},\cdots ,{\bar{H}}_{q_{x},Q_{y}-1},H_{q_{x},Q_{y}}\}diag\left(A_{P_{x}}^{H},\cdots ,A_{P_{x}}^{H},A_{P}^{H}\right), \end{array}$$

and ${\bar{H}}_{q_{x},n}=\left\{\left(R_{s}-\Phi_{x}^{1-q_{x}}\Theta^{*}\Phi_{y}^{1-n}R_{s}^{*}\Phi_{y}^{n-1}\Phi_{x}^{P_{x}-1}\Phi_{x}^{q_{x}-1}\right),\cdots ,\Phi_{x}^{q_{x}-1}\Phi_{y}^{Q_{y}-1}R_{s}\Phi_{y}^{1-n}\Phi_{x}^{1-q_{x}}\right\}$, $H_{q_{x},Q_{y}}$$=(R_{s}-\Phi_{x}^{1-q_{x}}\Theta^{*}\Phi_{y}^{1-Q_{y}}R_{s}^{*}\Phi_{y}^{Q_{y}-1}\Theta \Phi_{x}^{q_{x}-1})$

Based on the definition in (13), we can prove that the FO-ISD matrix $R^{f}$ has the following property.

**Theorem 1.**  Consider a URA consisting of M×N sensors, where both the x and y directions are separated by half a wavelength. By performing partial difference operation for each row rectangular subarray, we can form the FO-ISD matrix $R^{f}$ as in (13). Then, if $P_{x}P_{y}>K$, $Q_{x}Q_{y}>K$, $Q_{x},Q_{y}>1$, the rank of $R^{f}$ is equal to the number of coherent signals.

**Proof.**  See the Appendix A. ☐

#### 3.2.3. Forward Backward ISD (FB-ISD) Method

In this part, using the FB processing, we develop the FB-ISD method as follows.

As in (14), we can calculate that [20]

(15) $$\begin{array}{c} J_{\left(\left(Q_{y}+1\right)Q_{y}/2-1\right)P_{x}+P_{x}P_{y}}A_{1}=J_{\left(\left(Q_{y}+1\right)Q_{y}/2-1\right)P_{x}+P_{x}P_{y}}diag(A_{P_{x}}^{*},\cdots ,A_{P_{x}}^{*},A_{P}^{*})\\ \;=diag\left(J_{P_{x}}A_{P_{x}}^{*},\cdots ,J_{P_{x}}A_{P_{x}}^{*},J_{P_{x}P_{y}}A_{P}^{*}\right)=diag\left(A_{P_{x}}\Phi_{x}^{1-P_{x}},\cdots ,A_{P_{x}}\Phi_{x}^{1-P_{x}},A_{P}\Theta^{*}\right)\\ \;=A_{1}diag\left(\Phi_{x}^{1-P_{x}},\cdots ,\Phi_{x}^{1-P_{x}},\Theta^{*}\right), \end{array}$$

where $A_{1}=diag\left(A_{P_{x}}^{*},\cdots ,A_{P_{x}}^{*},A_{P}^{*}\right)$. Combining (14) and (15), we can get the backward spatial differencing matrix of $R_{q_{x}}$ as

(16) $$\begin{array}{cc} R_{q_{x}}^{b} & =J_{P_{x}P_{y}}R_{q_{x}}^{*}J_{\left(\left(Q_{y}+1\right)Q_{y}/2-1\right)P_{x}+P_{x}P_{y}}\\ & =A_{P}\Theta {\left\{{\bar{H}}_{q_{x},1},{\bar{H}}_{q_{x},2},\cdots ,{\bar{H}}_{q_{x},Q_{y}-1},H_{q_{x},Q_{y}}\right\}}^{*}diag\left(\Phi_{x}^{P_{x}-1},\cdots ,\Phi_{x}^{P_{x}-1},\Theta \right)A_{1}^{H}. \end{array}$$

Then, the FB-ISD matrix can be defined as

(17) $$\begin{array}{c} R^{fb}=\frac{1}{2Q_{x}}\sum_{q_{x}=1}^{Q_{x}}\left(R_{q_{x}}+J_{P_{x}P_{y}}R_{q_{x}}^{*}J_{\left(\left(Q_{y}+1\right)Q_{y}/2-1\right)P_{x}+P_{x}P_{y}}\right). \end{array}$$

**Remark 1.**  Comparing FO-ISD and FB-ISD, $R_{q_{x}}$ can extract all the information below the diagonal line, while $R_{q_{x}}^{*}$ includes the information above the diagonal line. Thus, FB-ISD use more data information than FO-ISD. Besides, due to the increased number of smoothing sub-matrices, FB-ISD can achieve a further improved performance than FO-ISD, including accuracy and resolution.

#### 3.2.4. Summary of FO-ISD and FB-ISD Methods

From (13) and (17), both FO-ISD and FB-ISD are proposed using the partial spatial differencing process for each row rectangular subarray. In fact, as described in Section 3.2.1, to improve the information utilization of the sample covariance matrix, we can first divide the URA into $Q_{y}$ column rectangular subarrays. Then, using the ISD scheme in Section 3.2.2 and Section 3.2.3, we can develop the corresponding FO-ISD matrix $R_{c}^{f}$ and FB-ISD matrix $R_{c}^{fb}$. In this case, the final ISD matrices can be set as

(18) $$\begin{array}{c} R_{0}^{f}=\left\{R^{f},R_{c}^{f}\right\},R_{0}^{fb}=\left\{R^{fb},R_{c}^{fb}\right\}. \end{array}$$

Clearly the final ISD matrices are more effective than $R^{f}$ and $R^{fb}$, due to the use of more data information. Then, the summary of proposed methods can be described as Algorithm 1.

  **Algorithm 1:** FO-ISD and FB-ISD for 2-D DOA estimation of coherent signals      **Input**   : **x**(t) = **As**(t) + **z**(t), t = 1, 2, …, *L*      **Output**: Estimated 2-D DOAs $\left(\theta_{k},\alpha_{k}\right),k=1,2,\ldots ,K$1 Calculate the sample covariance matrix of $x_{q_{x}x}\left(t\right)$ as ${\hat{R}}_{q_{x}x}=1/L\sum_{t=1}^{L}x_{q_{x}x}\left(t\right)x_{q_{x}x}^{H}\left(t\right)$;2 Construct the $q_{x}$-th spatial differencing matrix by performing partial difference-operation on the matrix ${\hat{R}}_{q_{x}x}$ as ${\hat{R}}_{q_{x}}$;3 Extract the information above diagonal submatrices as ${\bar{R}}_{1}=\left[{\bar{R}}_{1,N},{\bar{R}}_{1,N-1},\cdots ,{\bar{R}}_{1,N-P_{y}+1},{\bar{R}}_{1,1}\right]$ and compute its backward smoothing matrix ${\bar{R}}_{1}^{b}$;4 Build the FO-ISD matrix as ${\hat{R}}^{f}=1/Q_{x}\sum_{q_{x}=1}^{Q_{x}}{\hat{R}}_{q_{x}}$;5 Compute the FB-ISD matrix as ${\hat{R}}^{fb}=1/\left(2Q_{x}\right)\sum_{q_{x}=1}^{Q_{x}}\left({\hat{R}}_{q_{x}}+J_{P_{x}P_{y}}{\hat{R}}_{q_{x}}^{*}J_{\left(\left(Q_{y}+1\right)Q_{y}/2-1\right)P_{x}+P_{x}P_{y}}\right)$;6 Similar with Step 1∼6, for the column rectangular subarrays, construct the column spatial differencing matrix as ${\hat{R}}_{c}^{f}$ and ${\hat{R}}_{c}^{fb}$;7 Compute the final ISD matrices as ${\hat{R}}_{0}^{f}=\left\{{\hat{R}}^{f},{\hat{R}}_{c}^{f}\right\},{\hat{R}}_{0}^{fb}=\left\{{\hat{R}}^{fb},{\hat{R}}_{c}^{fb}\right\}$;8 Perform singular value decomposition (SVD) operation on the final ISD matrices and use 2-D ESPRIT algorithm for 2-D DOA estimation [28].

**Remark 2.**  AF-SDS and AFB-SDS only use the data information of overlapped smoothing subarrays, while the proposed scheme can extract all the data information of each row or column rectangle subarrays. Besides, both FO-ISD and FB-ISD only perform the difference-operation on the auto-correlations and the cross-correlations are kept unchanged. In this case, both the differencing submatrices and cross-correlations can be used to suppress the effect of additive noise. Therefore, the ISD scheme can achieve a performance improvement due to less information loss.

**Figure 3:**
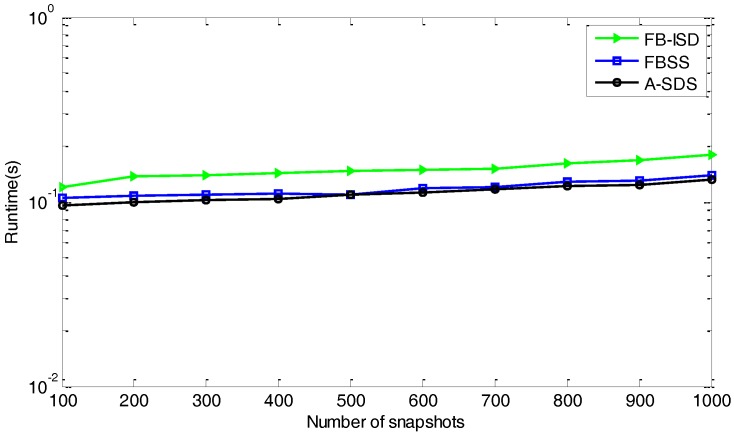
Comparison of runtime for relevant methods.

**Remark 3.**  FO-ISD and FB-ISD almost have the same computational complexity, which includes the formation of ISD matrices, SVD operation, and eigenvalue decomposition (EVD) operation. To avoid the increase of computational complexity, we can form the ISD matrices by using the sample covariance matrix of received signals, the cost of which is about $LM^{2}N^{2}+{\left(P_{x}P_{y}\right)}^{2}\left(P_{x}P_{y}+Q_{y}\left(Q_{y}+1\right)/2\right)+2K^{3}$. Then, similar to the proposed methods, we can also get the covariance matrix of the sliding windows for the spatial smoothing technique [17] or A-SDS method [27] by the same way, the cost of which is about $LM^{2}N^{2}+{\left(P_{x}P_{y}\right)}^{3}+2K^{3}$. So the proposed methods can achieve performance improvement with slightly higher computations. To be clear, we show the runtime of relevant methods in Figure 3 . We can see that FB-ISD has a heavier runtime load than that of FBSS and A-ISD.

**Remark 4.**  As described in Section 3.2, the proposed methods are developed for ideal sensors without any mutual coupling. Then, just like the methods in [29,30], the ISD scheme is also suitable for direction finding with unknown mutual coupling. However, when considering the array imperfections, the ISD scheme will fail due to the breakdown of array response matrices.

### 3.3. Cramér-Rao Bound (CRB)

As described in Section 2, according to [31], the CRB can be obtained as

(19) CRB=σ22LReDHΠA⊥D⊕R^sT−1,

where $D=\left[\frac{\partial a_{1}}{\partial \theta_{1}},\cdots ,\frac{\partial a_{K}}{\partial \theta_{K}},\frac{\partial a_{1}}{\partial \phi_{1}},\cdots ,\frac{\partial a_{K}}{\partial \phi_{K}}\right]$, ${\hat{R}}_{s}=\left[\begin{array}{cc} R_{s} & R_{s}\\ R_{s} & R_{s} \end{array}\right]$, $\Pi_{A}^{⊥}=I_{MN}-A{\left(A^{H}A\right)}^{-1}A^{H}$, $a_{k}$ is the *k*th column of A, *k* = 1, ⋯, *K*.

## 4. Simulation Results

We now evaluate the estimation performance of the ISD methods through in-depth numerical experiments. We assume the number of sensors is M=N=9. The wavelength of transmitted signals is set as 1*m* and the estimation performance is examined over 500 Monte Carlo trials. To evaluate the performance, the root-mean-square-error (RMSE) can be defined as

(20) $$\begin{array}{c} \mathrm{RMSE}=\sqrt{\frac{1}{2\bar{N}K}\sum_{i=1}^{\bar{N}}\sum_{k=1}^{K}\left[{\left(\alpha_{k}-{\hat{\alpha }}_{k}^{\left(i\right)}\right)}^{2}+{\left(\theta_{k}-{\hat{\theta }}_{k}^{\left(i\right)}\right)}^{2}\right]}. \end{array}$$

where $\bar{N}$ denotes the total independent trials and ($\alpha_{k}$, $\theta_{k}$) and (${\hat{\alpha }}_{k}$, ${\hat{\theta }}_{k}$) represent the true and estimated 2-D DOAs of the *k*-th signals, respectively.

### 4.1. Effectiveness Evaluation

**Figure 4:**
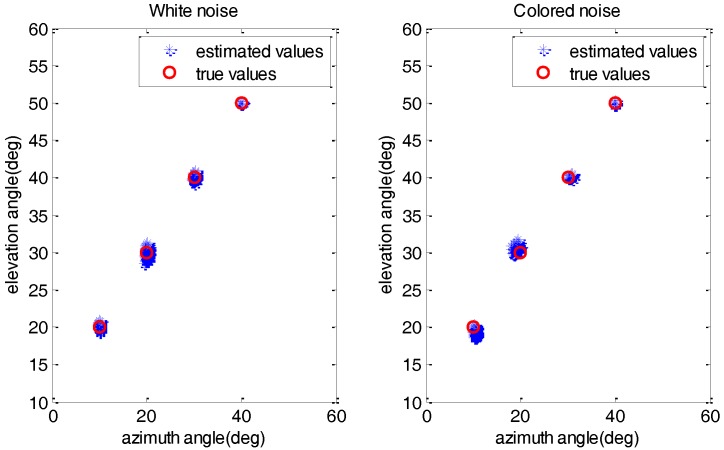
The estimated 2-D DOAs of FB-ISD method with 200 Monte Carlo trials.

In this experiment, we examine the effectiveness of the FB-ISD method for Gaussian white noise and colored noise, respectively. The colored noise is of a second-order autoregressive (AR) model with coefficients a=[1,−0.7,0.6][11,12,13,14,15,16]. The size of the subarrays are $P_{x}=P_{y}=6$ and the signals are located at $\alpha =[10^{{}^{\circ}},20^{{}^{\circ}},30^{{}^{\circ}},40^{{}^{\circ}}]$, $\theta =[20^{{}^{\circ}},30^{{}^{\circ}},40^{{}^{\circ}},50^{{}^{\circ}}]$. The number of snapshots is *L* = 300 and the signal to noise ratio (SNR) is 15 dB. Figure 4 shows the estimation results of FB-ISD with 200 Monte Carlo trials in white and colored noise conditions, respectively. As expected, all the 2-D DOAs can be estimated effectively and accurately. Besides, FB-ISD with white noise performs better than that of colored noise, especially for the first two signals.

**Figure 5:**
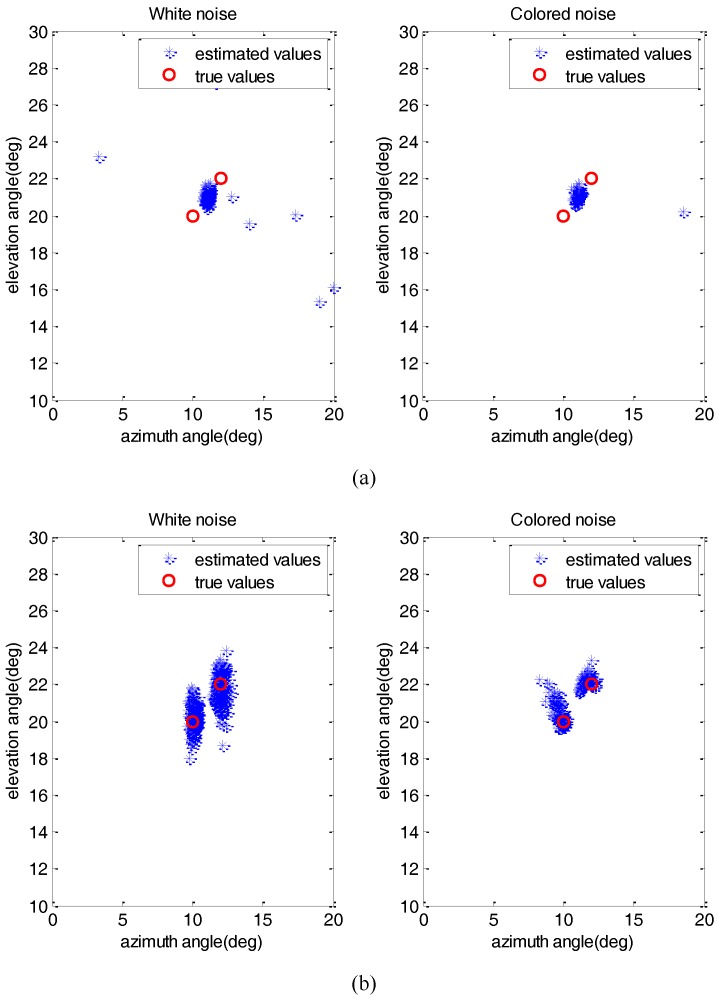
Comparison of resolution ability. (**a**) A-SDS method, (**b**) FB-ISD method.

Then, we compare the resolution ability by resolving two closely located signals in white and colored noise conditions, where the signals are located at $\alpha =[10^{{}^{\circ}},12^{{}^{\circ}}]$, $\theta =[20^{{}^{\circ}},22^{{}^{\circ}}]$, the SNR is 10 dB, and the number of snapshots is 200. Figure 5 describes the estimated DOAs of FB-ISD and A-SDS with 200 Monte Carlo trials. We can observe that FB-ISD can resolve the two signals successfully by using the difference-operation, while A-SDS fails to distinguish the signals.

### 4.2. RMSE Performance in the Case of White Noise

In this experiment, we examine the performance of the proposed methods versus SNR and the number of snapshots in the white noise condition. Here we assume the signal locations as $\alpha =[10^{{}^{\circ}},20^{{}^{\circ}},30^{{}^{\circ}}]$, $\theta =[20^{{}^{\circ}},30^{{}^{\circ}},40^{{}^{\circ}}]$, and we compare the proposed methods with other existing methods, including the forward only spatial smoothing (FOSS) method, FBSS method [16], and A-SDS method [27] ($p_{a}=3$). Moreover, the CRB is provided for comparison.

**Figure 6:**
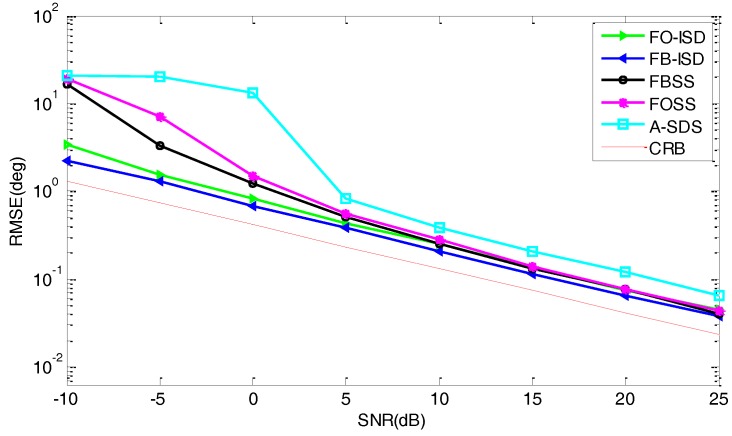
RMSE curves versus the SNR in the white noise condition.

*Performance versus SNR:* Figure 6 shows the RMSE versus SNR in the white noise condition, where we assume $P_{x}=P_{y}=7$ and L=300. It is observed that, the performance of FB-ISD is better than that of other methods due to the full use of data information. Since the partial difference-operation has less information loss than that of A-SDS, the performance of FB-ISD is much better, especially in the low SNR condition. Then, FB-ISD performs a little better than FO-ISD due to the FB processing. We also see that the RMSE curve of FB-ISD is very close to the CRB. In addition, combining with Figure 3, we can conclude that the proposed methods have a better performance but also higher computations.

**Figure 7:**
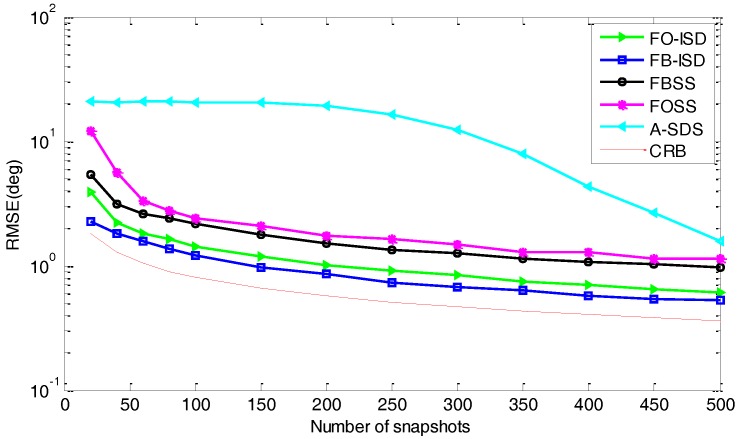
RMSE curves versus the number of snapshots in the white noise condition.

*Performance versus the number of snapshots*: Here, we evaluate the performance in terms of the number of snapshots, where the SNR is 0 dB and $P_{x}=P_{y}=7$. As shown in Figure 7, when the number of snapshots is small, FB-SMS still outperforms other methods and also matches closely to the CRB due to the use of more data information. Then, A-SDS performs much worse for the small number of snapshots, and the reason is that the information loss caused by difference-operation becomes greater with the decrease of snapshots. Besides, just like Figure 6, since spatial smoothing based methods suffer from aperture loss, all these methods cannot converge to the CRB in the limit for very high SNR condition.

### 4.3. RMSE Performance in Case of Colored Noise

In this experiment, we focus on the performance analysis of the proposed methods in a colored noise condition, where the performance for the white noise and colored noise conditions is also compared systematically.

**Figure 8:**
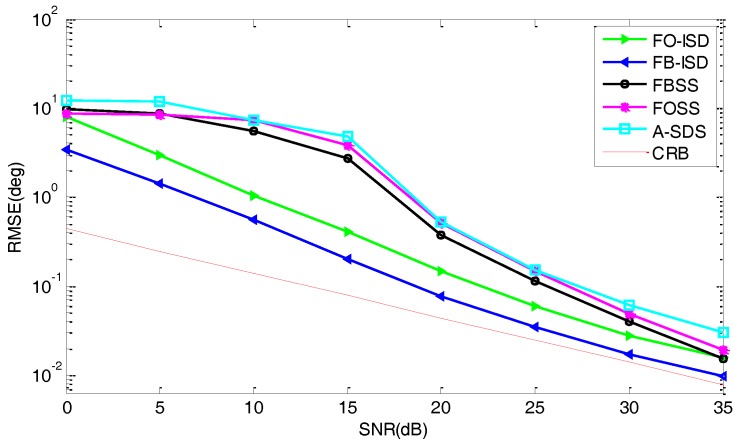
RMSE curves versus the SNR in the colored noise condition.

*Performance versus SNR*: Figure 8 presents the RMSE curves in terms of SNR in the colored noise condition, where the parameters are the same as Figure 7 except for the SNR varying from 0 dB to 35 dB. From Figure 8, we can see that the performance of FB-ISD is better than those of methods in [16,27]. Then, in the colored noise condition, FB-ISD and FO-ISD perform much better than others due to the partial difference-operation. Compared with Figure 6, we can summarize that the proposed scheme can suppress the effect of colored noise more effectively, and the reason is that the colored noise covariance matrix has significant values for diagonal and non-diagonal elements. Then, compared with A-SDS, we can also reach the following conclusion: FB-ISD can improve the performance greatly by only by performing the difference-operation on auto-correlations, for both colored noise and white noise.

**Figure 9:**
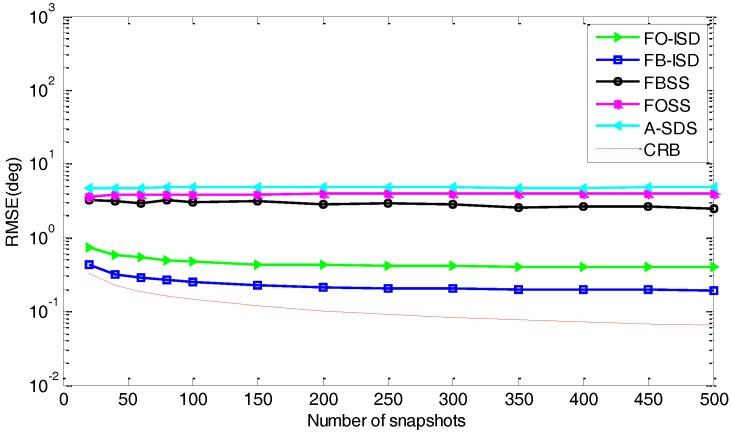
RMSE curve versus the number of snapshots in the colored noise condition.

*Performance versus the number of snapshots:* Figure 9 shows the RMSE against the number of snapshots in the colored noise condition, where the parameters are the same as Figure 7. As described in Figure 9, the performance of FB-ISD is superior to the other methods. Then, compared to Figure 7, the major distinction is that the performance for these methods is not much affected by the number of snapshots in the colored noise condition. The reason is that the information loss caused by difference-operation can also change with the number of snapshots. Besides, the proposed methods still have better performance, especially for the small number of snapshots.

## 5. Conclusions

In this paper, we have proposed an ISD scheme for 2-D DOA estimation of coherent signals with URAs, including FO-ISD and FB-ISD methods. By extracting all the data information of each row or column rectangular subarrays, we only performed the difference-operation on the auto-correlations, while the cross-correlations were kept unchanged. In this case, the reconstructed submatrices can use more data information of the sample covariance matrix and also suppress the effect of additive noise more effectively. Then, both the FO-ISD and FB-ISD methods were developed using the spatial smoothing submatrices. Simulation results demonstrated that, compared with other recently spatial smoothing and spatial differencing techniques, the performance of proposed methods was superior in white or colored noise conditions, in terms of accuracy and resolution ability.

## Appendix A

Combining (13) and (14), we have

(A1) $$\begin{array}{c} R^{f}=\left(1/Q_{x}\right)\sum_{q_{x}=1}^{Q_{x}}(A_{P}\{{\bar{H}}_{q_{x},1},{\bar{H}}_{q_{x},2},\cdots ,{\bar{H}}_{q_{x},Q_{y}-1},H_{q_{x},Q_{y}}\}diag(A_{P_{x}}^{H},\cdots ,A_{P_{x}}^{H},A_{P}^{H})) \end{array}$$

In the case of $rank\left(A_{P}\right)>K$, the rank of $D^{f}$ can be simplified as

(A2) $$\begin{array}{c} rank\left(R^{f}\right)=rank\left(\frac{1}{Q_{t}}\sum_{q_{t}=1}^{Q_{t}}\left\{{\bar{H}}_{q_{x},1},{\bar{H}}_{q_{x},2},\cdots ,{\bar{H}}_{q_{x},Q_{y}-1},H_{q_{x},Q_{y}}\right\}\right), \end{array}$$

Considering that the rank of a matrix is unchanged by a permutation of its column, we can rewrite $rank\left(D^{f}\right)$ as

(A3) $$\begin{array}{cc} rank\left(R^{f}\right)= & rank\left(\left\{\left(R_{s}-\Theta^{*}\sum_{q_{x}=1}^{Q_{x}}\Phi_{x}^{1-q_{x}}R_{s}^{*}\Phi_{x}^{q_{x}-1}\Phi_{x}^{P_{x}-1}\right),\cdots ,\right.\right.\\ & \left.\left.\left(R_{s}-\Theta^{*}\Phi_{y}^{1-Q_{y}}\sum_{q_{x}=1}^{Q_{x}}\Phi_{x}^{1-q_{x}}R_{s}^{*}\Phi_{x}^{q_{x}-1}\Phi_{x}^{P_{x}-1}\Phi_{y}^{1-Q_{y}}\right)\right\}\right), \end{array}$$

Using

(A4) $$\begin{array}{c} \sum_{q_{x}=1}^{Q_{x}}\Phi_{x}^{1-q_{x}}R_{s}^{*}\Phi_{t}^{q_{x}-1}=CC^{H},C=diag\left(p^{*}\right)A_{Q_{x}}^{H},R_{s}=pp^{H}, \end{array}$$

we have

(A5) $$\begin{array}{c} rank\left(R^{f}\right)=rank\left(\left\{\left(R_{s}-\Theta^{*}CC^{H}\Phi_{x}^{P_{x}-1}\right),\cdots ,\right.\right.\left.\left.\left(R_{s}-\Theta^{*}\Phi_{y}^{1-Q_{y}}CC^{H}\Phi_{y}^{Q_{y}-1}\Phi_{x}^{P_{x}-1}\right)\right\}\right), \end{array}$$

where $\Theta =\Phi_{y}^{P_{y}-1}\Phi_{x}^{P_{x}-1}$. Then, by assuming $F=\left\{p^{*},\Phi_{x}^{1-P_{x}}C,\cdots ,p^{*},\Phi_{x}^{1-P_{x}}\Phi_{y}^{1-Q_{y}}C\right\}$ and $G\;=\;\left\{I_{K},-\Phi_{y}^{1-P_{y}},\cdots ,I_{K},-\Phi_{y}^{1-P_{y}}\right\}$, the rank of $D^{f}$ can be represented as

(A6) $$\begin{array}{cc} rank\left(R^{f}\right) & =rank\left(Gblkdiag\left(F\right)blkdiag\left(F\right)\right)=rank\left(Gblkdiag\left(F\right)\right)=rank\left(F\right)\\ & =rank\left\{C,\cdots ,\Phi_{y}^{1-Q_{y}}C\right\}=rank\left(diag\left(p^{*}\right){\left(A_{Q_{x}}\circ A_{Q_{y}}\right)}^{H}\right), \end{array}$$

From (A6), since both $A_{Q_{x}}$ and $A_{Q_{y}}$ are Vandermonde matrices, the rank of $R^{f}$ is equal to *K*, if $rank\left(A_{Q_{x}}\circ A_{Q_{y}}\right)>K$.

Thus, we can conclude that, in the case of $rank\left(A_{P_{x}}\circ A_{P_{y}}\right)>K$ and $rank\left(A_{Q_{x}}\circ A_{Q_{y}}\right)>K$, the rank of $R^{f}$ is equal to the number of sources. That is, if $P_{x}P_{y}>K$, $Q_{x}Q_{y}>K$, $Q_{x},Q_{y}>1$, the rank of $R^{f}$ is equal to the number of coherent signals.

This completes the proof. ☐
